# The influence of mobile navigation design on older adults cognitive load, affective responses, and digital self-efficacy: mixed-methods study

**DOI:** 10.3389/fpsyg.2026.1851466

**Published:** 2026-06-08

**Authors:** Shining Jin, Bihua Liu, Tingyun Luo

**Affiliations:** School of Design, Fujian University of Technology, Fuzhou, China

**Keywords:** cognitive load, digital ageism, digital inclusion, gerontechnology, mixed methods, mobile navigation, older adults, psychological empowerment

## Abstract

**Objective:**

Drawing on the Stimulus-Organism-Response framework, this mixed-methods research examines how specific mobile navigation architectures, namely complexity and diversity, shape older adults' cognitive load, affective states, and enduring digital self-efficacy.

**Methods:**

The study utilized a two-phase design. Phase 1 involved a cross-sectional survey of 285 community-dwelling older adults, applying Partial Least Squares Structural Equation Modeling to test parallel mediation pathways. Phase 2 conducted a within-subject usability experiment with 25 participants evaluating five common mobile navigation paradigms, pairing real-time emotion tracking via the PANAS scale with granular behavioral metrics.

**Results:**

The PLS-SEM analysis indicated that high navigation complexity significantly drives up cognitive load (β = 0.111, *p* < 0.05) and dampens positive affect (β = −0.233, *p* < 0.001). In contrast, moderate design diversity boosted positive emotional responses (β = 0.330, *p* < 0.001) without triggering cognitive overload. Most notably, an exploratory hierarchical cluster analysis in Phase 2 suggested a potential performance- affect paradox: a subgroup of “cautious learners” took the longest to complete navigation tasks but reported the highest levels of positive affect and perceived control.

**Conclusions:**

These findings highlight the limitations of relying solely on the traditional efficiency-first paradigm in usability evaluation. For older adults, establishing a psychological sense of control frequently outweighs mere interaction speed. To advance sustainable digital inclusion, designers should move beyond purely reductive interfaces and instead design supportive environments that accommodate behavioral heterogeneity and foster true cognitive engagement.

## 1 Introduction

The expansion of digital technologies alongside global population aging has intensified concerns about how smart device use affects the mental health and social adaptation of older adults (Yang et al., 2025; Stanford and Just, 2025). While excessive use of digital technologies among younger populations is often associated with social isolation and poorer mental health, moderate and purposeful use among older adults can reduce depression, anxiety, and loneliness (Wen et al., 2023). It can also support social connection, access to healthcare resources, and independent living (Bertocchi et al., 2026). Within this process of digital adaptation, mobile interface navigation (Li and Luximon, 2020), as a core component of human-computer interaction, represents a critical access point through which older adults gain technological benefits.

A critical bottleneck in current age-friendly design is the frequent conflation of navigation complexity with navigation diversity. Navigation plays a pivotal role in shaping older adults' perception of usability and directly influences their adoption of new technologies (Li and Luximon, 2020). Older adults' declines in fluid intelligence, including reduced working memory capacity, slower information processing, and weakened spatial orientation, increase older users' sensitivity and vulnerability to navigation structure (Levy et al., 2021; Salthouse, 2019). However, guided by traditional usability heuristics, designers often resort to purely reductive interfaces that strip away functions to achieve superficial simplicity, in an attempt to mitigate the aforementioned navigation difficulties (Knowles and Hanson, 2018). While these surface-level usability issues are frequently observed, the deeper cognitive and interaction processing demands placed on older adults remain largely overlooked, leaving specific usability and graphic design challenges underexplored (Petrovčič et al., 2018). While structural complexity in the form of deep, text-heavy hierarchies imposes cognitive barriers that exacerbate older adults' navigation difficulties, navigational diversity achieved through flexible pathways and multimodal feedback serves as a compensatory mechanism, research is gradually focusing on the influence of navigation structure on user experience (Nagata et al., 2025; Siddique et al., 2026).

Current age-friendly design practices often emphasize simplification, guided by an efficiency-first logic that reduces operational barriers by minimizing interaction steps and enlarging visual elements (Tantra and Murthi, 2026). Although this efficiency-driven paradigm is effective in simplifying task execution and reducing instant usability frictions (Fisk et al., 2009), it may not be sufficient to foster deep digital engagement in older adults, reducing task difficulty alone does not necessarily enhance psychological empowerment (Gerich and Weber, 2019). In both interactive and work-related contexts, tasks that are achievable yet moderately challenging are more likely to strengthen individuals' sense of competence, perceived control, and long-term self-efficacy (Sherman and Seth, 2021). In digital environments, psychological empowerment can therefore be understood as a key mechanism linking interface design features to user performance outcomes (Xin et al., 2025; Hsieh et al., 2021; Zhou et al., 2022).

To systematically unpack these mechanisms, this study adopts the Stimulus-Organism-Response (SOR) theory as its foundational framework (Mehrabian and Russell, 1974; Jacoby, 2002). The SOR framework provides a scientifically sound causal structure to explain how objective mobile navigation architectures (Stimuli) uniquely trigger the intertwined cognitive and affective states (Organism) of older adults, ultimately shaping their digital self-efficacy (Response; Schreuder et al., 2016; Raj et al., 2023).

Grounded in the SOR framework, this study examines five common mobile navigation patterns, distinguishes between navigation complexity and navigation diversity, and assesses their differential effects on older users' cognitive and emotional responses. Specifically, the study aims to (1) examine how different navigation characteristics influence cognitive load and positive and negative affect; (2) test the mediating roles of cognitive load, affective responses, and perceived technology control in the relationship between navigation characteristics and digital self-efficacy; (3) identify behavioral and emotional differences among heterogeneous groups of older adults under different navigation conditions.

By incorporating emotional and psychological empowerment into human-computer interaction, this research extends the theoretical scope of age-friendly design. It also offers practical guidance for the evidence-based design of mobile applications that support the digital inclusion of older adults.

### 1.1 The SOR framework

Applied to older adults' interactions with mobile interfaces, this study operationalizes the SOR framework by defining the structural characteristics of mobile navigation, specifically complexity and diversity as the stimulus component (Li and Luximon, 2020; Cao et al., 2020). Building on this framework, the study integrates affective dimensions and psychological empowerment mechanisms into the interaction evaluation system by proposing two complementary pathways. The first is a cognitive pathway, in which navigation design influences digital self-efficacy through its impact on cognitive load (Liu and Li, 2024). This pathway captures how variations in navigation structure affect the cognitive systems of older adults, thereby shaping their digital self-efficacy beliefs. The second is an affective pathway, in which interface design translates cognitive resources required for task completion into intuitive emotional experiences during interaction. This pathway highlights how the subjective experience of interaction, rather than cognitive effort alone, contributes to users' sense of technology control and efficacy. These pathways provide a structured account of how navigation design influences both cognitive processing and emotional experience, and how these internal states jointly shape digital self-efficacy among older adults.

To clarify the complex psychological mechanisms underlying older adults' mobile interface interactions, this study uses the SOR framework to organize key constructs into a cohesive causal chain. Cognitive load and affective responses are viewed not as separate parallel processes but as intertwined internal states (Plass and Kalyuga, 2019) that mediate the effects of navigation design on perceived technology control and digital self-efficacy. This integrated approach moves beyond listing isolated constructs and establishes a unified theoretical explanation for how navigation design influences older adults' interaction experiences and outcomes.

### 1.2 Navigation pattern characteristics, cognitive load, and affective responses

Navigation complexity is defined as a structural barrier, characterized by hierarchical depth, excessive interaction steps, and high visual density. High-complexity mobile navigation is typically characterized by deep hierarchical structures and text-heavy menu systems, whereas low-complexity designs rely on more linear and streamlined navigation patterns that align with older adults' everyday experiences (Shi et al., 2021; Padhi et al., 2018). High levels of navigation complexity place substantial demands on limited working memory capacity, increasing the risk of cognitive overload and reducing perceived usability. When cognitive load exceeds manageable levels, older users' operational accuracy and trust in the system decline (Shi et al., 2021; Gomez-Hernandez et al., 2023). In contrast, low-complexity designs, such as linear navigation and simplified pathways, can reduce task completion time and decrease the need for assistance during interaction, thereby lowering cognitive burden (Lee et al., 2024; Zheng et al., 2024; Li and Luximon, 2022). However, excessively simplified designs may also remove opportunities for older users to engage meaningfully with tasks (Zhou et al., 2022; Gomez-Hernandez et al., 2023). When interaction becomes overly effortless, users may be less likely to experience a sense of achievement, which can negatively affect emotional responses, including technological anxiety, frustration, and irritability (An et al., 2024). By comparison, appropriately calibrated levels of complexity can effectively reduce the arousal of negative emotions and enhance positive emotions such as pleasure and competence, helping to strengthen self-efficacy (Zhu et al., 2025).

Navigation diversity refers to the range and variation of interaction paths and sensory modalities within a navigation system (Kuriakose et al., 2020). It offers flexible interaction pathways and multimodal feedback (e.g., combining visual, haptic, and auditory cues) that distribute information processing across multiple sensory channels rather than increasing structural complexity. Excessive diversity coupled with frequent switching between inconsistent structures, can exceed users' cognitive capacity and cause disorientation (Bayazit et al., 2018; Cao et al., 2024). Moderate diversity, by contrast, may compensate for age-related declines in single channel processing by engaging multiple sensory systems and promoting cognitive engagement (Diaz and Yalcinbas, 2021; Bates and Wolbers, 2014). When supported by a coherent structure, diversity enhances older adults' confidence in interface exploration and strengthens their digital self-efficacy (Lee et al., 2024; Zhou et al., 2023). When overly abundant and structurally incoherent, it raises cognitive load, reduces perceived control, and triggers negative affective responses such as anxiety and confusion (Huang and Hou, 2025; Carr et al., 2019). Moderate navigation diversity thus improves positive affect by supporting interaction fluency and a sustained sense of control.

Based on this reasoning, the following hypotheses are proposed:

H1: Mobile navigation complexity is significantly positively correlated with the cognitive load experienced by older users during operation.
H2: Mobile navigation diversity is significantly positively correlated with the cognitive load experienced by older users during operation.
H3: Mobile navigation complexity is significantly negatively correlated with the positive affect of older users.
H4: Mobile navigation diversity is significantly positively correlated with the positive affect of older users.

### 1.3 Affective responses, technology control, and self-efficacy

Affective responses and digital self-efficacy are key mechanisms influencing older adults' continued engagement with digital technologies (Jokisch et al., 2022; Liu and Li, 2024). Successful interaction experiences can evoke feelings of pleasure and accomplishment, which in turn enhance their willingness to explore new technologies (An et al., 2024). In addition, effective use of smart devices by older adults has been shown to reduce negative emotional states such as depression and anxiety, thereby contributing to improved psychological wellbeing (An et al., 2024; Qiu et al., 2024). Cognitive and affective processes are closely interrelated and mutually influential (Zhang et al., 2023). When cognitive load exceeds older users' available mental resources, it can impair task performance and lead to negative emotional responses, such as irritability and self-doubt (Mahjoob and Anderson, 2024). Conversely, when cognitive demands remain within manageable limits, users are more likely to experience cognitive fluency, which supports positive affect and reduces the likelihood of negative emotional reactions (Growney and English, 2025; Mahjoob and Anderson, 2024). Based on this reasoning, the following hypotheses are proposed:

H5: Cognitive load is significantly negatively correlated with the positive affect of older users during operation.
H6: Cognitive load is significantly positively correlated with the negative affect of older users during operation.

Within the SOR framework applied to older adult HCI, perceived technology control represents a critical mechanism for sustaining behavioral change and efficacy enhancement in older adults, with its psychological value far exceeding instrumental efficiency (Jokisch et al., 2022; Huang et al., 2025; Waki et al., 2025). Regarding individual affective responses, the speed of decline in self-efficacy due to negative technology experiences far outpaces the improvement brought about by positive experiences (Agogo and Hess, 2018). This suggests that minimizing negative emotional responses is particularly important in supporting sustained technology use among older adults (Liu and Wang, 2023). Only when older adults are able to establish a sense of technology control can moderately complex technology interactions translate into improved psychological outcomes and more positive emotional experiences (Liu and Wang, 2023). Mastery experience is widely recognized as a primary source of self-efficacy, and interaction designs that are intuitive, clear, and tolerant of user errors can facilitate the development of such experiences (Lozoya et al., 2022). In this way, well-designed navigation and functional design can strengthen older users' perceived control, enhance operational confidence, and support a transition from passive technology use to more active and autonomous engagement (Gomez-Hernandez et al., 2023; Amouzadeh et al., 2025). Accordingly, the following hypotheses are proposed, forming the final component of the theoretical model:

H7: The positive affect of older users is positively correlated with perceived control over mobile navigation.
H8: The negative affect of older users is negatively correlated with perceived control over mobile navigation.
H9: The positive affect of older adults is positively correlated with digital self-efficacy.
H10: The perceived control of older adults over mobile navigation is positively correlated with digital self-efficacy.

**Figure 1:**
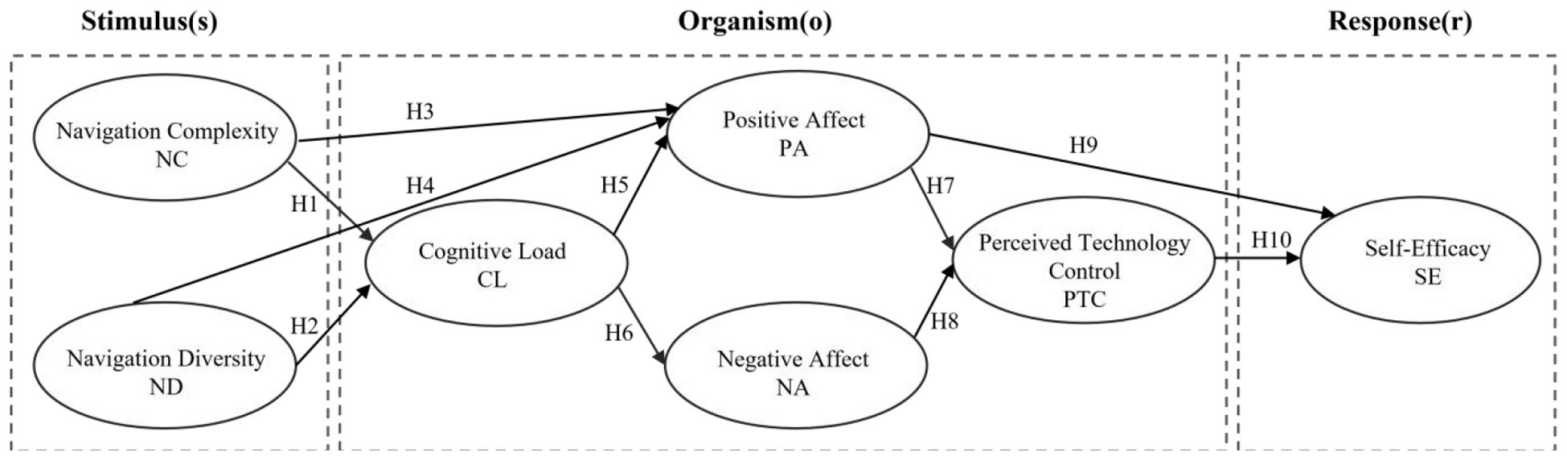
The proposed research model for hypotheses H1–H10.

Drawing upon the 10 hypotheses formulated above, we developed a structural model to investigate the mechanisms driving older adults' digital self-efficacy in mobile navigation. Grounded in the SOR framework, this model systematically links external interface stimuli, specifically navigation complexity and diversity, with users' internal organismic states, which include cognitive load, affective responses, and perceived technology control, to explain the ultimate behavioral response of digital self-efficacy. As depicted in Figure 1, the diagram visually represents the directional relationships among these constructs.

Each arrow in the model corresponds to a specific hypothesis ranging from H1 to H10, indicating the expected path of influence. By encapsulating these multi-stage interactions, the model provides a structured and comprehensive framework for analyzing how mobile navigation design shapes psychological empowerment and digital inclusion among older populations.

## 2 Materials and methods

This study adopts a mixed-methods design combining a questionnaire-based survey with structural equation modeling (SEM) and a controlled interaction experiment. Data were collected in Fujian Province, China, between January and December 2024. The research protocol was conducted in accordance with established academic ethics standards and received approval from the Human Research Ethics Committee of Fujian University of Technology.

### 2.1 Study 1: pre-testing

#### 2.1.1 Participants

For the pre-test, five older adult participants were recruited directly from the target population to complete one-on-one cognitive interviews. For the main formal survey, participants were recruited through older adult learning centers and day care centers across multiple communities in Fujian Province. Eligibility participants were required to meet two primary inclusion criteria: being aged 60 years or older and possessing prior experience using smartphones. A total of 285 valid responses were formally collected, resulting in an effective response rate of 94.14%. Detailed demographic characteristics of these respondents are reported in Table 1. To ensure methodological rigor, the sample size was justified based on established guidelines for PLS-SEM. According to the widely recognized 10-times rule (Hair et al., 2021), the minimum threshold is 30 (10 times the maximum number of structural paths directed at a specific construct, which is three in our model). Furthermore, referring to the more stringent inverse square root method proposed by Kock and Hadaya (2018) for PLS-SEM, a sample size of 160 is typically sufficient to detect path coefficients of 0.21 or greater with 80% power at a 5% significance level. Our actual sample size of 285 exceeds both rigorous thresholds, indicating sufficient statistical power.

**Table 1:** Demographic information of respondents.

Item	Category	Frequency	Percentage (%)
Gender	Male	144	50.5
	Female	141	49.5
Age	Between 60 and 64	133	46.7
	65–69 years old	101	35.4
	Over 70 years old	51	17.9
Education background	High school and below	70	24.6
	Junior college	140	49.1
	Undergraduate	65	22.8
	Graduate student or above	10	3.5
Older navigation experience	With	206	72.3
	Without	79	27.7

#### 2.1.2 Materials

Study 1 was designed to collect large-sample cross-sectional data. The questionnaire and measurement items were developed and refined based on existing literature (Table 2). The survey instrument consisted of two main sections: the first collected baseline demographic information, and the second measured the seven latent variables included in the theoretical model. All items were assessed using a seven-point Likert scale ranging from 1 (strongly disagree) to 7 (strongly agree).

**Table 2:** Measurement items and reliability test results.

Construct	Item	References
NC1.	The more steps I had to click to navigate through the APP, the more difficult I found it.	Wong et al., 2024; Carter et al., 2018; Sobrinho et al., 2024; Tajudeen et al., 2022
NC2.	The more choices I have to make in APP navigation, the more focused I need to be.	
NC3.	The more cumbersome the APP navigation steps, the more I felt my ability to keep up and the pressure to operate was great.	
ND4.	There were many different ways to navigate within the same APP, and it took me more time to get used to it.	Shimokihara et al., 2023; Shi et al., 2021; Wang et al., 2018
ND5.	The APP navigation style changes a lot, which makes me feel confused and easy to get lost.	
ND6.	Trying out different navigation options in the APP made me feel like my phone was improving.	
CL1.	When navigating the APP, it takes a lot of mental effort to find the features I want.	Kim et al., 2024; Gomez-Hernandez et al., 2023; Shimokihara et al., 2023
CL2.	I struggled to remember multiple steps to navigate the APP at once.	
CL3.	In the face of multiple APP navigation methods, I feel that the time is tight and I am afraid of not finishing the operation.	
CL4	When navigating the APP successfully, I found the process painless and effortless.	
CL5.	A variety of APP navigation operations, will make me feel that my ability to keep up with, the heart is very anxious.	
PA1.	When I successfully navigate my APP, I feel a sense of accomplishment.	Rodríguez-Martínez et al., 2023
PA2.	When I successfully find the desired function, I feel very happy and encouraged.	
PA3.	When the navigation function of the app operates smoothly, I will have more confidence in my ability to use the mobile phone.	
NA1	When I keep making mistakes in typing and can't find the navigation function, I will feel annoyed and frustrated.	Rodríguez-Martínez et al., 2023; Park et al., 2017
NA2.	When the navigation steps are too complicated, I will feel nervous and afraid that I might mess it up.	
NA3.	Whenever the navigation operation fails, I always feel very frustrated.	
PTC1.	Even if the navigation operation takes a little longer, as long as I can complete it independently, I feel that I have control over the navigation.	
PTC2.	I can independently figure out the usage of new navigation functions on my own, and I feel that I can control the navigation operations of the APP.	Shimokihara et al., 2023; Sobrinho et al., 2024
PTC3.	As long as I can successfully complete the navigation task, I feel in control of the APP navigation, even if the action is slow.	
PTC4.	When navigating the APP, I know exactly what to do next and feel in control.	
SE1.	I'm confident I can navigate the APP on my own and find what I'm looking for.	Wong et al., 2024; Shimokihara et al., 2023; Kim and Han, 2021
SE2.	Even if there is an operation error, I believe I can find a way to solve it.	
SE3.	Even if I haven't seen the APP navigation mode, I am confident that I can learn how to use it.	
SE4.	I think I have the ability to skillfully use the functions of the APP.	

#### 2.1.3 Procedure

To ensure content validity, two experts in age-friendly design first reviewed the initial questionnaire to evaluate the items' structural rationality, content relevance, and professional appropriateness. Subsequently, we recruited five older adult participants from the target population to complete a one-on-one cognitive interview-based pre-test. This pre-test focused on assessing the readability, intelligibility, and age-appropriateness of the wording, specifically aiming to identify ambiguous terms, overly complex sentence structures, or concepts inconsistent with older adults' daily expressive habits. Drawing on expert feedback alongside the cognitive interview results, several items in the Chinese version were revised to enhance comprehensibility for older respondents. Following these revisions, the finalized survey instrument was formally distributed to older adults for the main study.

#### 2.1.4 Statistical procedures

Statistical evaluation of the cross-sectional data was conducted applying Partial Least Squares Structural Equation Modeling (PLS-SEM). Specifically, SPSS 26.0 was used to assess internal consistency reliability, while SmartPLS 4.0 was utilized to evaluate the measurement model (convergent and discriminant validity) and the structural model.

To test the parallel mediation pathways, the significance of all 10 hypotheses (H1–H10) was evaluated using the bootstrapping method. Structural model assessment included explanatory power (*R*^2^), predictive relevance (*Q*^2^), multicollinearity (VIF), and overall fit via the Standardized Root Mean Square Residual (SRMR) and Normed Fit Index (NFI).

### 2.2 Study 2: within-subject experimental design of interaction navigation patterns

Study 2 employed a within-subject experimental design to examine how different mobile navigation patterns affect older adults' operational performance and real-time emotional responses. Through controlled interaction tasks, the study aimed to capture and measure fine-grained behavioral data and immediate affective fluctuations during user interaction.

#### 2.2.1 Participants

The experiment employed purposive sampling to recruit 25 older adult participants from public welfare smartphone training programs at community Elderly Academies in Fujian Province. The sample consisted of 13 males and 12 females, with an age distribution of 10 participants aged 60–64, 5 aged 65–69, 8 aged 70–74, and 2 aged 75 years or older. Due to the exploratory nature of this interactive experiment, specific education level data was not formally recorded. Recruiting digital novices from a uniform training background helped reduce variability in prior app experience, this purposive approach does not constitute a strict experimental control. In terms of testing the number of elderly users, referring to the research on usability testing and interactive system testing, it is found that 5–16 users are enough to find a reasonable number of problems in small and medium-sized systems (Faulkner, 2023; Nielsen, 1994; Woolrych and Cockton, 2001). Although the sample size of *N* = 25 limits the statistical power for drawing definitive between-group inferences, it remains valuable for conducting heuristic exploratory analyses to identify potential behavioral phenotypes of older users. Therefore, Study 2 did not attempt to generalize these statistical differences to the broader population, but rather positioned the cluster analysis as a preliminary exploratory step.

This study inclusion criteria were as follows: (1) aged 60 years or older; and (2) possessing basic smartphone operation experience. Exclusion criteria included diagnosed neurodegenerative diseases, psychological disorders, severe fine motor impairments of the hands, and uncontrolled major chronic conditions. All participants were screened prior to inclusion in the study. Given that participants were recruited from novice training programs, we explicitly operationalized this experience level through two detailed criteria. First, regarding app familiarity, participants typically used only fundamental applications, such as WeChat for basic communication and phone calls. Second, regarding usage frequency and skills, they possessed basic touch-screen operational abilities (e.g., tapping and swiping), but they had little to no prior experience independently navigating deep, multi-layered applications.

#### 2.2.2 Materials

The experimental device was a smartphone with a 6.4-inch display, capable of running all test applications smoothly. Data collection materials included an observation recording form used to document behaviors such as pauses, navigation errors, and verbal requests for assistance. Immediate emotional responses were measured using the Chinese version of the PANAS scale, employing a five-point Likert scale (1 = strongly disagree, 5 = strongly agree) to measure immediate emotional responses. Positive Affect (PA) and Negative Affect (NA) were computed as the summed scores of their respective items. A composite Positive Emotional Experience (PE) index was derived by subtracting the NA score from the PA score (Diener et al., 1999).

**Figure 2:**
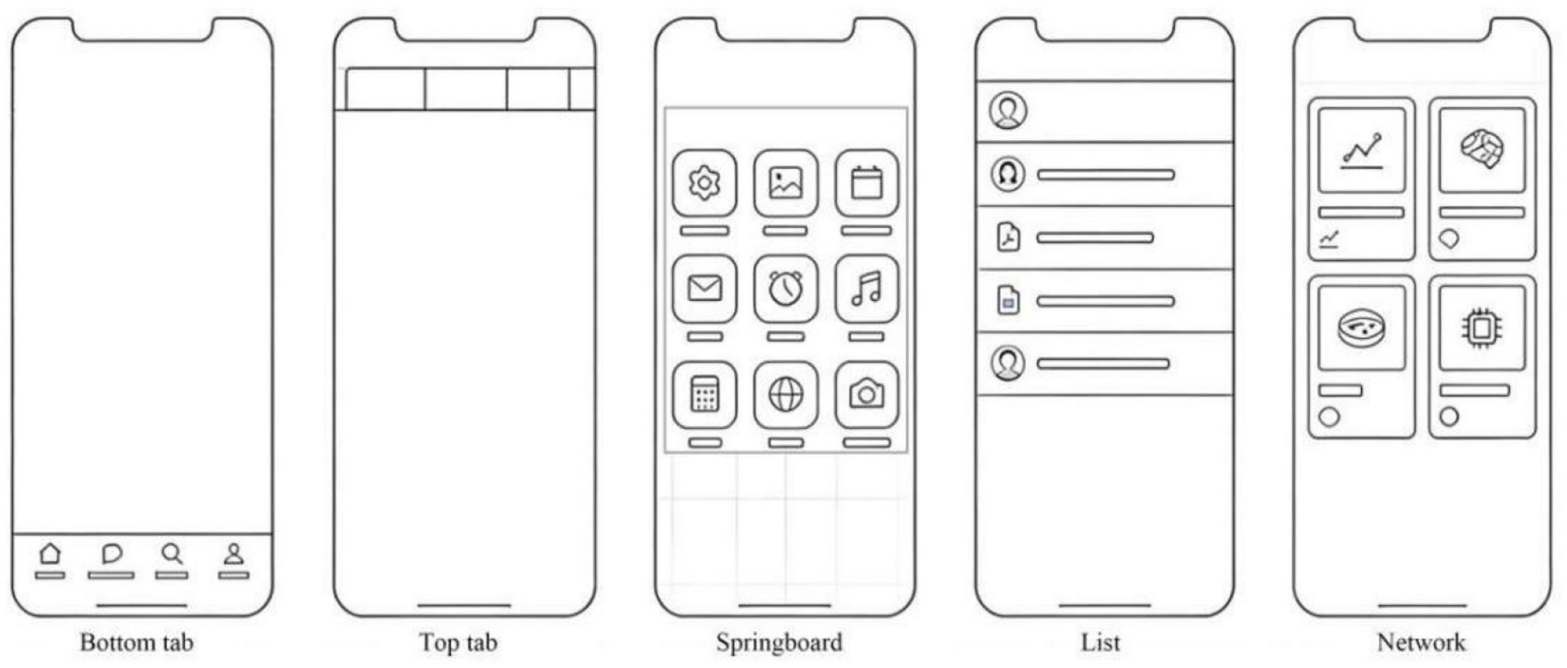
Examples of navigation design patterns used by older adult users (bottom tab, top tab, springboard, list, and network).

Based on a review of iOS and Android design guidelines, as well as commonly used applications, five representative mobile navigation patterns were identified: bottom tab, top tab, springboard, list, and network (Figure 2). To enhance ecological validity and avoid disconnect between abstract wireframes and the real-world usage scenarios of older adults, widely used applications among the older adults, namely WeChat, TikTok, and Toutiao Large, were selected as experimental platforms. These applications represent common usage contexts, including social communication, entertainment, and information access, respectively, and collectively cover the identified navigation patterns. For WeChat and TikTok, only visual elements were adjusted to improve accessibility, while the underlying navigation structures were retained. In contrast, the information-based application was adapted with a simplified and flattened navigation architecture to better suit older adult users interaction needs. Navigation complexity was operationalized in terms of the cognitive effort required to complete navigation tasks, measured along two dimensions: (1) information encoding modality and (2) number of operational steps. Navigation diversity was defined in terms of interaction flexibility and sensory richness, measured through (1) the variety of interaction or gesture types and (2) the range of multi-sensory feedback mechanisms. The relationship between the five navigation patterns and the two independent variables, along with corresponding experimental tasks, is presented in Table 3. This design aimed to ensure a clear manipulation of complexity and diversity while minimizing confounding effects between the two variables.

**Table 3:** Mobile navigation pattern and core independent variable dimension matching matrix.

Navigation/step no.	Navigation complexity	Navigation diversity	Task steps	Task difficulty
Bottom-tab [1]	Low	Low	1–2	Low
Top-tab [10]	Low	Low	2–3	Low
Springboard [5]	Low	High	1	High
List [2], [11], [12], [18]	High	Low	3–4	Low
Network [17]	High	High	4–6	Low

**Figure 3:**
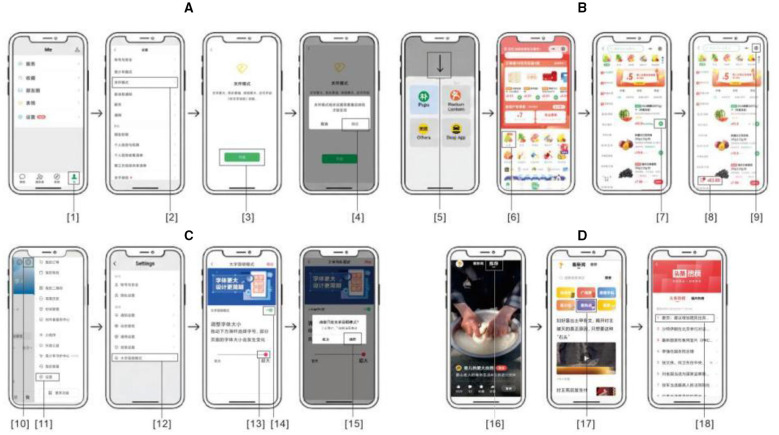
Task steps no. for WeChat task **(a)** [1–4], WeChat task **(b)** [5–9], TikTok task **(c)** [10–15], and Toutiao Large task **(d)** [16–18].

(1) Navigation complexity level classification: ≤ 3 operation steps with dual-modality (text and graphical) encoding, low complexity; ≥4 operation steps with single-modality encoding, high complexity. (2) Navigation diversity level classification: single interaction mode with visual-only feedback, low diversity; ≥2 interaction modes with multi-sensory feedback, high diversity. Detailed task steps are illustrated in Figure 3.

Task (a): Activating WeChat Senior Mode. Participants were instructed to navigate through the settings interface to enable senior mode. This task primarily involved bottom-tab and long-list navigation, requiring sequential menu exploration.

Task (b): Shopping within the WeChat mini-program ecosystem. Participants performed a simulated grocery shopping task by entering a designated mini-program, browsing network-based categories, selecting a specific fruit, adding it to the shopping cart, and exiting the interface. This task involved multiple navigation types, including springboard, network, and list navigation, and imposed relatively high cognitive demands.

Task (c): Switching TikTok to Senior Mode. Participants were required to access a hidden sidebar and navigate to deeper-level settings. In this mode, textual labels were minimized, requiring participants to rely primarily on icon recognition and spatial memory.

Task (d): Browsing Toutiao's hotlist. Participants navigated between top-tab categories and accessed detailed content within the hotlist (Figure 3).

#### 2.2.3 Procedure

A standardized operating procedure (SOP) was developed to ensure consistency across sessions. All interaction sessions were recorded using the smartphone's built-in screen recording function, enabling later millisecond-level replay analysis of interaction time and error patterns for each click. During the experiment, due to equipment constraints in the field setting, participants were assigned identification numbers chronologically based on their testing start times. All participants completed the designated navigation tasks in a strictly sequential, fixed order. Furthermore, to ensure high reproducibility and minimize researcher bias, a SOP was strictly enforced. Prior to each task, the researcher read the task instructions from a pre-defined, standardized script with a neutral tone. During task execution, researchers were prohibited from providing spontaneous guidance; standardized verbal prompts were only delivered if a participant explicitly requested help or remained inactive for an extended period. Each task session lasted approximately 15 min. To accommodate older participants physical stamina and reduce fatigue, the emotional assessment using the PANAS scale was administered by the researchers in a structured question-and-answer format. The total duration per participant, including task completion and emotional assessment, was approximately 20 min. This experiment was designed to simulate common daily tasks, comprising a total of 18 interaction steps. All interaction sessions were recorded using the smartphone's built-in screen recording function. Through controlled interaction tasks, fine-grained behavioral data and immediate affective fluctuations were captured and extracted. Task completion time (in seconds) was measured from the participant's initial screen touch to the successful execution of the final step. Error rate was calculated following, Chung et al. (2010) as [(*N*_ai_ – *N*_ri_)/*N*_ri_] × 100%, where *N*_ai_ represents the number of actual inputs and *N*_*ri*_ represents the number of required inputs. After completing the tasks, participants were assessed using the PANAS scale.

#### 2.2.4 Statistical procedures

Statistical analysis of all data collected was performed with SPSS 26.0. Hierarchical cluster analysis (HCA) was performed to identify heterogeneity between subjects using their overall task completion time and number of errors as variables. Given the sample sizes and non-normal distribution of the clustered groups, the Kruskal–Wallis *H* non-parametric test was used to evaluate intergroup differences.

## 3 Results

### 3.1 Study 1: pre-test

#### 3.1.1 Measurement and structural model assessment

The PLS-SEM measurement model was evaluated via preliminary tests of internal consistency, convergent validity, and discriminant validity. Table S1 in the Supplementary materials shows Cronbach's Alpha and CR for all constructs exceeded 0.70 and 0.75, respectively, confirming sufficient internal consistency. Following the suggestion of Fornell and Larcker (1981), convergent validity was verified as all standardized factor loadings exceeded 0.80 and all AVE exceeded 0.65. Discriminant validity was established via two criteria: the Fornell–Larcker criterion, where the square root of each construct's AVE exceeded all inter-construct correlations, and the HTMT criterion (Henseler et al., 2015), where all values were below the conservative 0.85 cut-off.

With a valid measurement model confirmed, we assessed the structural model's fit, explanatory relevance, and predictive relevance. Multicollinearity was absent, with all VIF values between 1.000 and 1.864, well-below the five threshold. The model showed acceptable overall fit: SRMR was 0.029, meeting the 0.08 requirement, and NFI was 0.916, exceeding the 0.90 threshold. Finally, the model demonstrated strong predictive relevance, explaining 49.3% variance in digital self-efficacy and 46.0% in cognitive load. For predictive relevance assessment, we followed Chin (1998) methodological recommendation to use the cross-validated redundancy index *Q*^2^, with a *Q*^2^ value greater than 0 indicating the structural model predictive relevance. All cross-validated redundancy *Q*^2^ values ranged from 0.182 to 0.455, all well-above zero, as detailed in Table 4.

**Table 4:** Model path analysis results.

Item	Hypothesis	β	*t*	*p*	VIF	Result
H1	NC –> CL	0.111	2.123	0.035^*^	1.290	Supported
H2	ND –> CL	0.084	1.482	0.139	1.290	Unsupported
H3	NC –> PA	−0.233	−4.500	0.000^**^	1.694	Supported
H4	ND –> PA	0.330	6.402	0.000^**^	1.393	Supported
H5	CL –> PA	−0.14	−1.174	0.241	1.864	Unsupported
H6	CL –> NA	0.03	0.263	0.793	1.000	Unsupported
H7	PA –> PTC	0.123	2.099	0.037^*^	1.364	Supported
H8	NA –> PTC	0.13	2.335	0.020^*^	1.364	Supported
H9	PA –> SE	0.296	5.891	0.000^**^	1.404	Supported
H10	PTC –> SE	0.374	7.009	0.000^**^	1.404	Supported

β, Standardized path coefficient; VIF, variance inflation factor. ^*^p < 0.05, ^**^p < 0.01. VIF < 5 indicate the absence of severe multicollinearity.

#### 3.1.2 Hypothesis testing results

**Figure 4:**
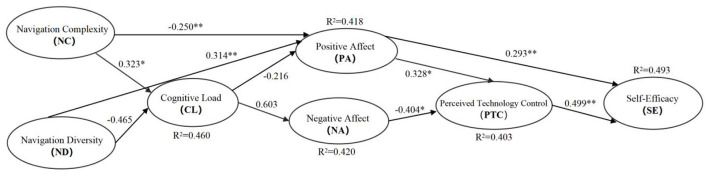
The result of the direct path. *p < 0.05, **p < 0.01.

Of the 10 research hypotheses proposed, seven (H1, H3, H4, H7, H8, H9, and H10) were supported, while H2, H5, and H6 were not statistically significant. The structural model results are shown in Figure 4.

(1) S → O: Navigation complexity and navigation diversity exhibited distinct effects on the cognitive load and affective responses of older users. Navigation complexity significantly positively increased cognitive load (H1: β = 0.111, *p* < 0.05) and significantly reduced positive affect (H3: β = −0.233, *p* < 0.001). In contrast, moderate navigation diversity significantly increased positive affect (H4: β = 0.330, *p* < 0.001), but did not significantly affect cognitive load (H2: β = 0.084, *p* = 0.139). These findings suggest that navigation complexity primarily functions as a cognitive burden, whereas appropriately designed navigation diversity may enhance emotional experience without substantially increasing cognitive demands (Diaz and Yalcinbas, 2021; Bates and Wolbers, 2014; Newcombe et al., 2023).

(2) O → R: Affective responses played a significant role in shaping perceived technology control. Positive affect was positively associated with perceived technology control (H7: β = 0.123, *p* < 0.05), whereas negative affect was negatively associated with perceived control (H8: β = −0.130, *p* < 0.05). However, the hypothesized relationships between cognitive load and affective responses (H5, H6) were not supported. This suggests that cognitive load and affective responses may operate as partially independent dual mechanisms (Mahjoob and Anderson, 2024) rather than as a single linear process.

(3) *R* (Perceived Technology Control and Self-Efficacy): At the response stage, both affective and control-related factors contributed to digital self-efficacy. Positive affect directly enhanced digital self-efficacy (H9: β = 0.296, *p* < 0.001), and perceived technology control also had a strong positive effect (H10: β = 0.374, *p* < 0.001). These results indicate that both emotional experience and perceived control are important drivers of older adults' confidence in using digital technologies (Zhu et al., 2025; Mannheim et al., 2019).

### 3.2 Study 2: within-subject experimental design of interaction navigation patterns

#### 3.2.1 Objective performance

Based on frame-by-frame analysis of recorded interaction sessions for the 25 participants, task completion time and error rates were extracted for each task. For Task (d), which featured a flattened network-list navigation structure and increased visual spacing, participants demonstrated the highest operational fluency, with the shortest average completion time (*M* = 12.10, *SD* = 5.95) and the lowest error rate (*M* = 0.20, *SD* = 0.41). Tasks (a) and (b), which involved springboard and network navigation, showed moderate completion times [Task (a): *M* = 18.60, *SD* = 8.16; Task (b): *M* = 16.45, *SD* = 6.25] and relatively low error rates [Task (a): *M* = 0.44, *SD* = 0.50; Task (b): *M* = 0.36, *SD* = 0.49]. These results may reflect participants' prior familiarity with similar interaction patterns in commonly used applications. In contrast, Task (c) involved a long interaction path of six steps, typical of deep hierarchical structures. Its senior mode's metaphorical design stripped away icon text labels, causing the longest average completion time (*M* = 35.10, *SD* = 11.60) and the highest error rate (*M* = 1.36, *SD* = 0.70). This finding suggests that each extra layer of navigational hierarchy is associated with significantly longer task completion times and higher error rates among older adults in our sample (Li and Luximon, 2020). It further indicates that navigation designs dependent on icon recognition and spatial memory may place greater cognitive strain on this population (Chung et al., 2025).

#### 3.2.2 Subjective affective responses

Across all tasks, participants reported a mean PA score of 3.75 and a mean NA score of 1.17, indicating generally PE and low levels of NA. Within the PA dimension, items such as “*determined*” and “*enthusiastic*” received the highest scores (Table 5). Observational data suggest that tasks with clear practical relevance (e.g., shopping or browsing news) were associated with higher engagement and willingness to explore. “*Inspired*” also received relatively high scores. These observations suggest that successful problem-solving during interaction may contribute to positive emotional experiences, including feelings of accomplishment and focus.

**Table 5:** PANAS statistics table.

(PA)	Items	Score	Mean	(NA)	Items	Score	Mean
	Interested	105	3.89		Distressed	34	1.26
	Excited	106	3.93		Upset	37	1.37
	Strong	95	3.52		Guilty	30	1.11
	Enthusiastic	113	4.19		Scared	34	1.26
	Proud	105	3.89		Hostile	29	1.07
	Alert	104	3.85		Irritable	28	1.04
	Inspired	108	4.00		Ashamed	38	1.41
	Determined	119	4.41		Nervous	32	1.19
	Attentive	74	2.74		Jittery	27	1.00
	Active	84	3.11		Afraid	27	1.00

#### 3.2.3 Cluster analysis

**Figure 5:**
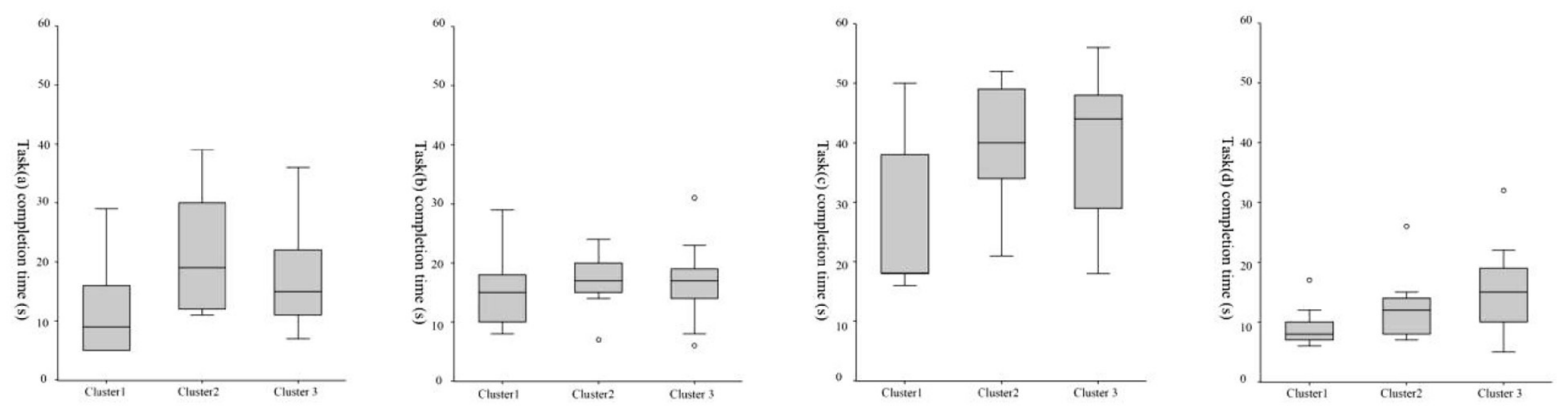
Task completion time and error rate across the three clusters. ° represent mild outliers (data points between 1.5 and 3 times the IQR).

**Figure 6:**
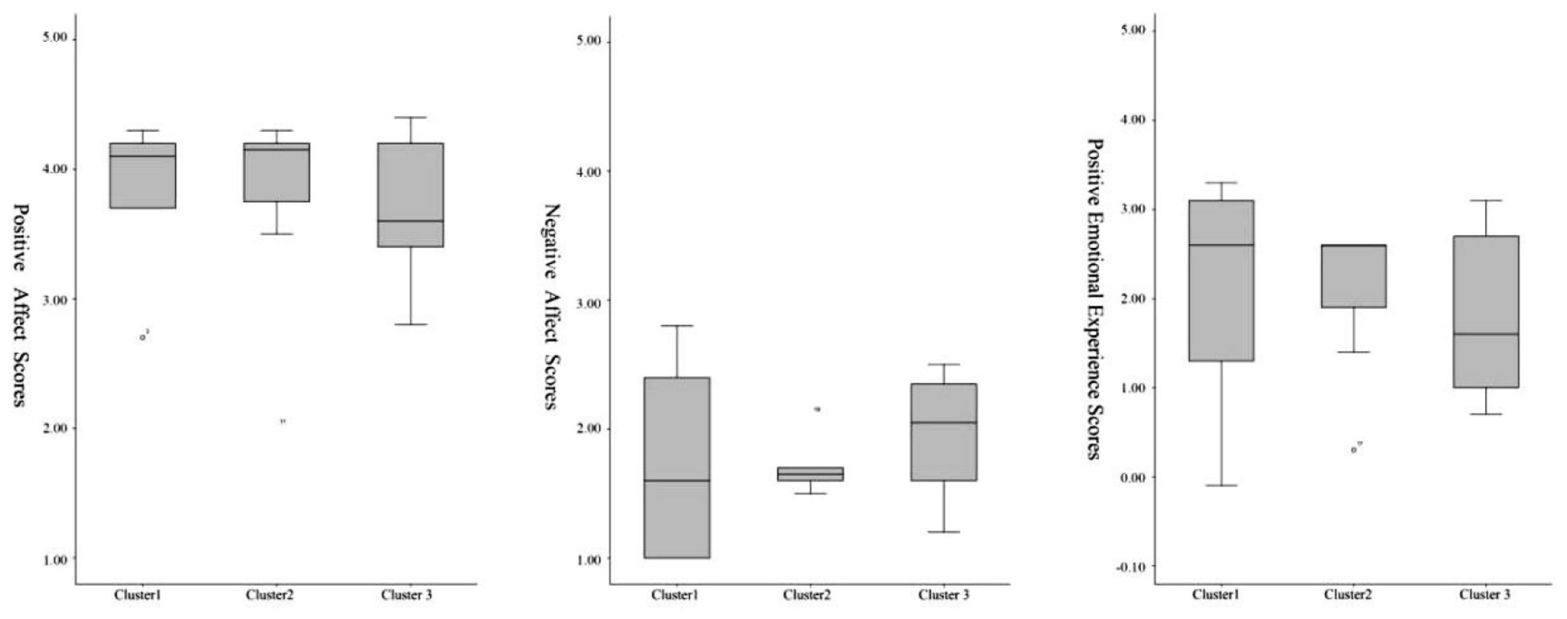
PANAS results across the three clusters. ° represent mild outliers (data points between 1.5 and 3 times the IQR).

The exploratory analysis suggested three potential user profiles with differing behavioral and emotional characteristics (Table 6, Figures 5, 6).

**Table 6:** Behavioral and affective comparisons across the three clusters.

Cluster	*N*	Task time		Error count		PA		NA		PE	
		Mean	SD	Mean	SD	Mean	SD	Mean	SD	Mean	SD
1	5	66.40	27.39	2.60	2.70	3.80	0.66	1.76	0.82	2.04	1.43
2	8	90.75	20.44	1.88	1.25	3.81	0.77	1.69	0.18	2.13	0.85
3	12	87.42	22.54	3.75	2.22	3.71	0.51	1.96	0.46	1.75	0.90

1. Cluster 1, *n* = 5: Skilled users. This group demonstrated the shortest average completion time (*M* = 66.4, *SD* = 15.52) and efficient error correction (*M* = 0.80, *SD* = 0.84). Their overall PE was moderate (*M* = 3.60, *SD* = 0.55). Although they reported high levels of PA (e.g., “*attentive*”, “*determined*”), they also exhibited relatively higher levels of negative affect (e.g., “*jittery*”, “*afraid*”), suggesting a state of focused but effortful engagement.

2. Cluster 2, *n* = 8: Cautious users. This group showed the longest average completion time (*M* = 90.75, *SD* = 10.36) but the lowest error count (*M* = 0.63, *SD* = 0.52). Despite lower efficiency, they reported the highest PE (*M* = 4.25, *SD* = 0.46), with particularly strong scores on “*proud*” and “*attentive*.” These findings suggest that careful task execution and successful learning experiences may be associated with higher perceived control and self-efficacy, even when performance efficiency is lower.

3. Cluster 3, *n* = 12: Frustrated users. This group exhibited moderate completion times (*M* = 87.42, *SD* = 18.25), but the highest error count (*M* = 3.25, *SD* = 1.60). They also reported the lowest PE (*M* = 2.42, *SD* = 0.67), with elevated scores on negative affect items such as “*upset*” and “*guilty*.” Observational data suggest that these participants experienced difficulty developing effective error-recovery strategies and were more likely to attribute difficulties to personal limitations.

The results indicate statistically significant differences in emotional experiences across clusters when facing bottom tab and springboard navigation (Table 7). Further exploratory analysis of navigation interaction indicated that participants in Cluster 2 tended to require more time using these two navigation modes.

**Table 7:** Kruskal–Wallis *H*-test results for clustering and navigation patterns.

Navigation	Cluster (mean ±SD)			*p*
	Cluster1 (*n* = 5)	Cluster2 (*n* = 8)	Cluster3 (*n* = 12)	
Bottom-tab [1]	2.00 ± 1.73	6.50 ± 3.42	2.50 ± 2.15	0.004^**^
Top-tab [10]	3.60 ± 1.82	9.00 ± 5.71	5.92 ± 4.48	0.125
Springboard [5]	2.60 ± 1.67	7.25 ± 5.18	1.67 ± 1.15	0.002^**^
Network [17]	4.00 ± 4.58	2.00 ± 0.76	2.08 ± 1.56	0.254
Network [6]	3.60 ± 2.19	4.38 ± 1.77	6.58 ± 3.48	0.097
List [18]	4.00 ± 4.24	3.38 ± 2.26	4.67 ± 2.50	0.611
List [2]	4.20 ± 3.70	5.63 ± 1.92	5.25 ± 3.84	0.750
List [12]	8.40 ± 6.84	7.88 ± 3.44	6.33 ± 3.11	0.569
List [11]	3.00 ± 1.22	4.00 ± 3.21	4.92 ± 3.29	0.479

The numbers in brackets (e.g., [1], [5], [10]) correspond to the specific task interaction steps illustrated in Figure 3. ^**^p < 0.01.

**Figure 7:**
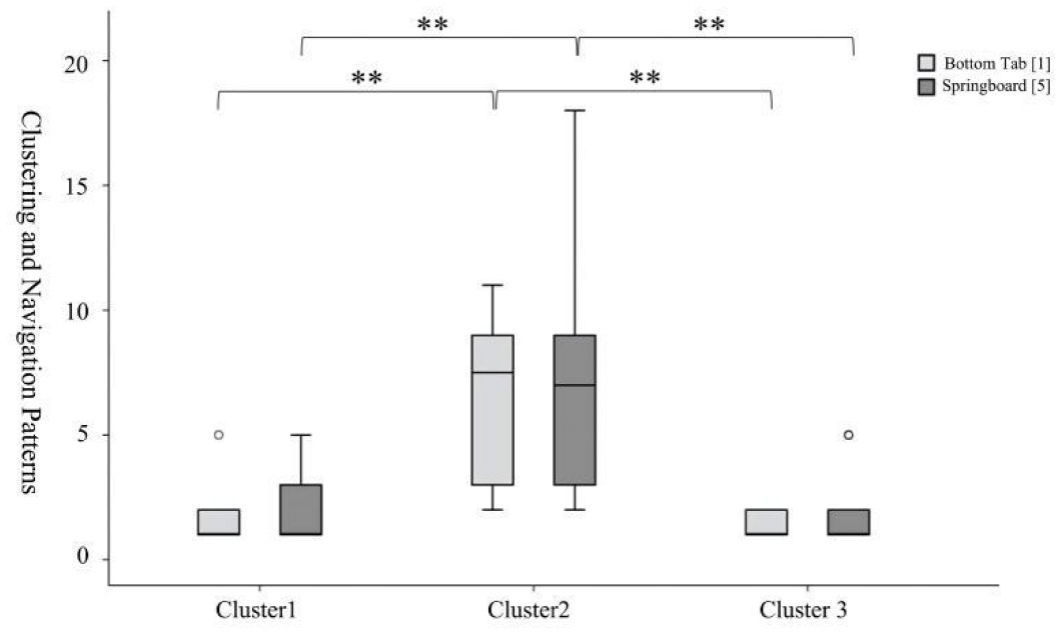
Box plots illustrating behavioral performance differences across the three user clusters under specific navigation patterns (Bottom Tab vs. Springboard). **indicate significant pairwise differences (*p* < 0.01), highlighting that Cluster 2 required significantly more time to complete tasks than both Cluster 1 and Cluster 3 in the Bottom Tab and Springboard navigation modes. ° represent mild outliers.

User feedback on navigation interaction preferences (Figure 7) suggested distinct behavioral patterns across the three clusters. Cluster 1 showed strong adaptability to springboard and bottom tab interfaces, favoring direct and efficient interaction methods, which corresponded with consistently shorter task completion times. Cluster 2 despite having the highest PE scores, required more time when using icon-based interfaces such as bottom tab and springboard navigation. This pattern suggests a learning-oriented interaction approach, characterized by reliance on structured guidance and a higher tolerance for errors. Cluster 3 demonstrated moderate performance with springboard navigation but exhibited longer completion times and signs of frustration when interacting with text-heavy top tab interfaces. This indicates greater difficulty with cognitively demanding navigation structures.

## 4 Discussion

The results indicate differentiated effects of navigation complexity and navigation diversity on the cognitive load and emotional responses of older users. High-complexity navigation designs, particularly those involving deep hierarchical text-based structures (Chandra and Manjunath, 2010), were associated with increased cognitive overload and reduced PE. Such designs require users to maintain and update multi-level navigation paths in working memory, which may exceed available cognitive resources and hinder efficient interaction. In contrast, navigation diversity, when implemented at a moderate level, did not significantly increase cognitive load and was associated with higher levels of positive affect. This finding differs from prior studies suggesting that increased visual complexity, high information density, deep hierarchical structures negatively affect usability and cognitive processing (Zhou et al., 2022), particularly among older adults. One possible explanation is that excessive reliance on a single visual channel may overload working memory (While et al., 2024), whereas moderate diversity across interaction modalities may distribute cognitive processing demands. In this sense, navigation diversity may function as a form of cognitive support, reducing reliance on internal memory processes and facilitating more efficient information processing.

The exploratory cluster analysis and SEM results jointly suggest a potential performance-affect paradox in older adults' digital interaction (Khan et al., 2026; Liu and Li, 2024). Conventional usability frameworks typically take efficiency, namely shorter task completion times and lower operational error rates, as the core indicators of user experience and satisfaction (Zeng et al., 2016). However, the exploratory cluster analysis in Study 2 observed that the “cautious learning” subgroup in this sample took the longest total task completion time yet reported the highest positive emotional experience after task completion. In parallel, the Study 1 SEM results did not identify a statistically significant direct effect of increased cognitive load on negative or positive affective responses. These findings do not support a linear sequential chain where cognitive load directly dictates affective states in this study model. Instead, it suggests that cognitive load and affective responses operate as partially independent mechanisms during older adults' digital interactions. Crucially, this emotional independence does not imply that cognitive overload is harmless; eye-tracking evidence confirms that excessive navigation depth objectively exacerbates cognitive strain and impairs older users' online trust (Wang et al., 2026). These findings suggest that age-appropriate design should not focus solely on complexity, but rather on supporting a positive cycle of moderate challenge, perceived control, and achievement feedback, through which older users can strengthen self-efficacy within cognitively manageable limits.

The findings further clarify the differentiated influence pathways through which navigation complexity and navigation diversity influence cognitive load and digital self-efficacy among older users. The negative effect of high navigation complexity appears to operate primarily through two partially independent pathways: deep, single-modality navigation on one hand increasing cognitive load, and on the other hand, it is associated with weakened positive affect, which subsequently may undermine perceived control and reduce self-efficacy. In contrast, the positive effect of moderate navigation diversity does not operate through cognitive load, but begins with the activation of positive affect. Navigation designs that combine multiple interaction modes and multi-sensory feedback (Kuriakose et al., 2020) can stimulate positive emotional responses, which in turn strengthen perceived control and enhance digital self-efficacy. Among these pathways, perceived technological control appears to be the strongest proximal driver of digital self-efficacy (Luan and Li, 2026; Martins et al., 2025). Taken together, these results suggest that navigation complexity functions primarily as a risk factor through cumulative cognitive burden, whereas moderate navigation diversity functions as a protective factor through affective engagement and empowerment.

This study has several limitations that should be addressed in future research. First, regarding the questionnaire development in Study 1, while content validity and readability were ensured through expert reviews and cognitive interviews with five older adults, future studies could employ a larger and more socio-demographically diverse pilot sample to capture an even broader spectrum of linguistic nuances before formal data collection. Second, the sample size in the controlled experiment of Study 2 (*N* = 25) is relatively small. Consequently, the hierarchical cluster analysis and subsequent non-parametric group comparisons should be interpreted with caution. The identification of user groups (e.g., “cautious learners”) serves as a heuristic, exploratory analysis of behavioral phenotypes rather than definitive proof of absolute statistical differences across the broader aging population. Future research with larger experimental cohorts is required to statistically validate these user groups. Furthermore, to maximize ecological validity, Study 2 embedded navigation tasks within diverse real-world applications (WeChat, TikTok, and Toutiao). This design inherently introduces confounding factors such as app familiarity, task semantics, and functional difficulty, which could not be completely eliminated in our study. Future studies should employ standardized, brand-agnostic high-fidelity prototypes to isolate specific navigation variables more strictly. Third, although the combined cross-sectional design and controlled experiment capture immediate cognitive, emotional, and behavioral responses, this approach cannot account for the long-term, dynamic evolution of digital self-efficacy in real-world settings. Future studies could adopt longitudinal designs or *in-situ* observations to better understand how these processes evolve over time. Finally, cognitive load in this study is primarily measured using subjective scales and behavioral performance indicators. Although, these measures are widely used, they do not capture underlying neural processes. Future research could incorporate multimodal neurophysiological objective measurement methods, such as eye tracking and functional near-infrared spectroscopy to provide more direct evidence of cognitive processing. Integrating subjective assessments, behavioral data and neurophysiological measures would enable triangulation across data sources, offering a more comprehensive understanding of how navigation design influences cognitive load and, ultimately, supporting the development of more effective age-appropriate design.

## 5 Conclusion

This study examines the roles of navigation complexity and navigation diversity in shaping cognitive load, emotional response and digital self-efficacy among older users (Li and Luximon, 2020). The findings highlight a divergence between performance and emotional outcomes, where lower operational efficiency may coexist with higher PE. The study also identifies two distinct pathways within the SOR framework, providing empirical evidence for how different navigation design features influence user experience and self-efficacy (Mehrabian and Russell, 1974; Raj et al., 2023).

At the theoretical level, the contributions of this study can be summarized in three aspects. First, it extends the application of the SOR framework to older adults' digital interaction by linking interface-level design features to psychological outcomes, including digital self-efficacy, building on the work of Cao et al. (2020) and Raj et al. (2023), who also applied the SOR framework to explore the links between interface design features and user psychological and behavioral outcomes in digital interaction contexts. Second, it distinguishes between navigation complexity and navigation diversity as separate design dimensions, clarifying their different effects on cognitive load, affective responses, and perceived control. It challenges the conventional emphasis on efficiency as the primary indicator of emotional experience and perceived capability in age-appropriate design (Gerich and Weber, 2019; Sherman and Seth, 2021). Overall, the findings suggest that effective navigation design for older users should not focus solely on reducing complexity, but also consider how interaction structures can support positive emotional engagement and perceived control, thereby contributing to greater digital confidence and inclusion.

At the practical level, this study provides guidance for optimizing navigation design in mobile application for older users. Rather than relying solely on minimalist or automated design approaches, navigation design should account for the cognitive characteristics and psychological needs of older adults. Informed by this study findings, design strategies would benefit from prioritizing flat navigation structures, supported by dual-channel coding of text and graphics, while limiting hierarchical depth (e.g., to no more than two levels where possible). Additionally, based on the observed cognitive strain, it is advisable to minimize pure icon-based navigation without text labels and deeply hidden interface elements, such as sidebars. For users adopting a more cautious learning approach, interfaces could support them by providing clear guidance, error tolerance, and sufficient time for task completion. For users experiencing greater difficulty, design features such as visual anchors and accessible exit mechanisms may help reduce frustration and facilitate recovery from errors. Importantly, within cognitively manageable limits, our findings suggest that allowing for independent exploration and trial-and-error can be beneficial, as these processes may contribute to users' sense of control and self-efficacy.

## Data Availability

The raw data supporting the conclusions of this article will be made available by the authors, without undue reservation.
