# Supplementary material for: The influence of mobile navigation design on older adults cognitive load, affective responses, and digital self-efficacy: mixed-methods study
Source: Front Psychol. 2026 Jun 8;17:1851466. doi: 10.3389/fpsyg.2026.1851466 (PMC13284980; doi:10.3389/fpsyg.2026.1851466)
Supplement: Supplementary file 1 [file Data_Sheet_1.pdf]

Table S1. Reliability, convergent validity, and discriminant validity of the measurement model.

Convergent validity analysis results.

**construct**	**Item**	**Std.**	**AVE**	**CR**
Navigation Complexity	NC1.	0.903	0.869	0.952
	NC2.	0.882		
	NC3.	0.904		
Navigation Diversity	ND4.	0.880	0.848	0.944
	ND5.	0.889		
	ND6.	0.988		
Cognitive Load	CL1.	0.896	0.836	0.962
	CL2.	0.894		
	CL3.	0.879		
	CL4.	0.894		
	CL5.	0.938		
Positive affect	PA1.	0.939	0.812	0.928
	PA2.	0.855		
	PA3.	0.858		
Negative affect	NA1	0.913	0.828	0.935
	NA2.	0.870		
	NA3.	0.888		
Perceived Technology Control	PTC1.	0.933	0.822	0.949
	PTC2.	0.903		
	PTC3.	0.875		
	PTC4.	0.911		
Self-Efficacy	SE1.	0.949	0.814	0.946
	SE2.	0.885		
	SE3.	0.879		
	SE4.	0.894		

Fornell–Larcker test results.

	**NC**	**ND**	**CL**	**PA**	**NA**	**PTC**	**SE**
Navigation Complexity	**0.932**						
Navigation Diversity	-0.469	**0.921**					
Cognitive Load	0.540	-0.613	**0.914**				
Positive affect	-0.513	0.562	-0.541	**0.901**			
Negative affect	0.531	-0.544	0.600	-0.514	**0.91**		
Perceived Technology Control	-0.429	0.423	-0.516	0.535	-0.572	**0.907**	
Self-Efficacy	-0.504	0.506	-0.557	0.559	-0.572	0.656	**0.902**

HTMT ratio test results.

	**NC**	**ND**	**CL**	**PA**	**NA**	**PTC**	**SE**
Navigation Complexity	-						
Navigation Diversity	0.496	-					
Cognitive Load	0.566	0.646	-				
Positive affect	0.546	0.602	0.573	-			
Negative affect	0.564	0.58	0.633	0.552	-		
Perceived Technology Control	0.451	0.447	0.541	0.571	0.607	-	
Self-Efficacy	0.532	0.537	0.584	0.598	0.609	0.692	-

Note: NC=Navigation Complexity;ND=Navigation Diversity;CL=Cognitive Load;PA=Positive affect;NA=Negative affect;PTC=Perceived Technology Control;SE=Self-Efficacy.
